# COVID-19 Testing and Vaccine Willingness: Cross-Sectional Survey in a Culturally Diverse Community in Sydney, Australia

**DOI:** 10.1089/heq.2021.0171

**Published:** 2022-12-28

**Authors:** Julie Ayre, Danielle M. Muscat, Olivia Mac, Carys Batcup, Erin Cvejic, Kristen Pickles, Hankiz Dolan, Carissa Bonner, Dana Mouwad, Dipti Zachariah, Una Turalic, Yvonne Santalucia, Tingting Chen, Gordana Vasic, Kirsten McCaffery

**Affiliations:** ^1^ Faculty of Medicine and Health, Sydney Health Literacy Lab, School of Public Health, The University of Sydney, Sydney, Australia.; ^2^ Western Sydney Local Health District, Sydney, Australia.; ^3^ Nepean Blue Mountains Local Health District, Sydney, Australia.; ^4^ South Western Sydney Local Health District, Sydney, Australia.

**Keywords:** health literacy, culture, community health promotion, health knowledge promotion, COVID-19

## Abstract

**Objective:**

The current study examined patterns in COVID-19 testing and vaccination intentions across multiple language groups in Greater Western Sydney, Australia.

**Methods:**

Participants completed a cross-sectional survey available from March 21 to July 9, 2021 in Sydney, Australia. Surveys were available in English or translated (11 languages). Participants could complete surveys independently or with support from bilingual staff. Logistic regression models using poststratification weighted frequencies identified factors associated with testing and vaccination intentions.

**Results:**

Most of the 708 participants (88%, *n*=622) were not born in Australia; 31% reported that they did not speak English well or at all (*n*=220); 70% had no tertiary qualifications (*n*=497); and 41% had inadequate health literacy (*n*=290). Half (53.0%) reported willingness to get a COVID-19 vaccine if recommended to them (*n*=375); 18% were unwilling (*n*=127), and the remainder unsure (29%, *n*=205). These proportions varied significantly by language group (*p*<0.001). Participants were more likely to be unwilling/hesitant if they were female (*p*=0.02) or did not use Australian commercial information sources (*p*=0.01). Concerns about side effects (30.4%, *n*=102) and safety (23.9%, *n*=80), were key reported barriers to vaccination. Most participants reported high testing intention (77.2%, *n*=546), with differences observed across language groups (*p*<0.001). The most frequently reported barrier to testing was concerns about infection at the clinic (26.1%) followed by concerns that testing was painful (25.3%).

**Conclusion:**

Different language groups have unique and specific needs to support uptake of COVID-19 testing and vaccination. Health services must work collaboratively with culturally and linguistically diverse communities to provide tailored support to encourage COVID-19 testing and vaccination.

## Introduction

In 2021, vaccination became the main COVID-19 management strategy to enable countries to ease restrictions, open up the economy, and protect those who are most vulnerable from serious consequences of the virus.^1^ Australian COVID-19 vaccine rollout began in February 2021. During and beyond the rollout, testing for COVID-19 symptoms (e.g., fever, cough, sore throat) and subsequent contact tracing of positive cases continues to be another important management strategy.

Australia has taken a state-based and phased approach to COVID-19 vaccine rollout, with eligibility focusing initially on frontline health care workers, older people, Aboriginal and Torres Strait Islander people, and people with specified preexisting underlying medical conditions.^2^ Two vaccines were available at the time of the study: Pfizer (Comirnaty) and AstraZeneca (Vaxzevria), although the supply of Pfizer was much more limited. Though both are widely used and accepted internationally in countries such as the United Kingdom, the AstraZeneca vaccine has drawn media attention due to a very small increased risk of very serious side effects, and reports of poorer vaccine effectiveness compared with Pfizer.^3^ This, coupled with changing official advice about Pfizer as the “preferred vaccine” for certain age groups likely reduced community willingness to get the AstraZeneca vaccine in the first half of 2021, which slowed the rollout because it was the only option available to many community members at that time.^4^

Australian nationally representative surveys suggest that during the COVID-19 vaccine rollout, the proportion of Australians who agreed that they would get a COVID-19 vaccination when it became available ranged from 68% to 73% between April and June 2021.^5,6^ Before vaccines became available, our Australia-wide surveys suggested that in April and November 2020, the main barriers to COVID-19 vaccination were concerns about the vaccines' safety, distrust of the government or the vaccine, and needing more information.^7–9^ No differences in vaccine intention were observed for people who spoke a language other than English at home, although people with lower health literacy and less education had lower vaccine intentions. This finding is consistent with another nationally representative survey (sample recruited August 2020).^10^

Since April 24th, 2020, all Australians have been encouraged to get tested for COVID-19 if they experience even mild symptoms. However, nationally representative surveys estimate that the proportion of Australians reporting that they would definitely get a COVID-19 test if they had mild respiratory infection symptoms is relatively low, ranging between 45% and 51% between December 2020 and June 2021.^5,6^ Our survey in November 2020 highlighted that the most common barriers to testing included having symptoms but not believing it was COVID (e.g., hayfever) (15%), preferring to self-isolate (13%), feeling that symptoms were not severe enough (11%), and concern that the test was painful (10%).^11^

The above research provides useful data on COVID-19 attitudes and intentions in *general* Australian samples. However, only a small proportion of participants had inadequate health literacy or spoke a language other than English at home. This limits our capacity to understand the needs of Australian communities that are typically understudied and underserved, such as those that are culturally and linguistically diverse. This is critically important; in other countries such as the United Kingdom and the United States, research indicates that COVID-19 testing rates and vaccine acceptability are often lower for people in culturally and linguistically diverse communities,^12,13^ with calls to prioritize testing and vaccination efforts for these groups and ensure equitable health outcomes.^14,15^

The current study aimed to fill this research gap by describing patterns in COVID-19 testing intentions and vaccine willingness in a culturally and linguistically diverse sample in Sydney, Australia, during the period March 21 to July 9, 2021; and to identify key barriers to COVID-19 testing and vaccine willingness within these groups.

## Methods

### Study design

This study used a cross-sectional survey design. The study was approved by Western Sydney Local Health District Human Research Ethics Committee (Project No. 2020/ETH03085).

### Setting

Participants were recruited from March 21 to July 9, 2021. During this period, the COVID-19 vaccine rollout had begun across Australia although supply of the vaccine was limited and the “phased” nature of the rollout meant that many were not yet eligible to receive the vaccine, particularly those less than 40 years of age. As such, rollout was slower than anticipated in NSW. Daily cases of community transmission in NSW ranged from 0 to 45 during this time.^16^ This level of community transmission represented a low level of risk, which is reflected by the public health authorities' removal of most COVID-19 restrictions from March until late June. Restrictions across Greater Sydney were then reinstated on June 23rd. These included limitations on the number of people allowed to visit a household, maximum number of people in an exercise class, and reduced seating capacity for outdoor events.^17^

On the day the survey closed (July 9th), the NSW daily case count was 45, and NSW Health announced stay-at-home orders for Greater Sydney.^18^ The survey was closed at this time despite some recruitment targets not reached so that results could be more readily interpreted.

### Participants

Participants were eligible to take part if they were 18 years of age or over and spoke one of the following as their main language at home: Arabic, Assyrian, Croatian, Dari, Dinka, Hindi, Khmer, Chinese, Samoan/Tongan (combined as one language “group”), or Spanish. We selected these ten language groups through iterative discussions with multicultural health staff, with the aim of providing broad coverage across different global regions, groups with varying average levels of English language proficiency (based on 2016 Australian census data),^19^ varying access to non-English materials, and varying degrees of reading skill in their main language spoken at home (Supplementary Appendix SA2). Each of the language groups selected was an important group within the Greater Western Sydney region (Western Sydney Local Health District, South Western Sydney Local Health District, Nepean Blue Mountains Local Health District).

Participants were recruited through bilingual Multicultural Health staff and Health Care Interpreter Service staff. Multicultural Health staff recruited participants through their existing networks, community events, and community champions. Health Care Interpreter Service staff recruited participants at the end of a medical appointment (in public health facilities [e.g., hospitals and community health centers]). Potential participants were offered two means of taking part: completing the survey themselves online (available in English or translated), or bilingual staff or an interpreter-entered responses into the survey platform on the participants' behalf. To ensure consistency in the phrases used for assisted survey completion, translated versions of the survey were provided to the bilingual staff and interpreters.

### Survey design

Surveys were available in English or translated, and hosted on the web-based survey platform Qualtrics. Demographic survey items included age, gender, education, years living in Australia, main language spoken at home, self-reported English language proficiency, reading proficiency in language spoken at home, access to the internet, access to smartphones, chronic disease, and a single-item health literacy screener.^20^

Vaccine willingness was assessed by asking “*If a COVID-19 vaccine were recommended for you, would you get it?*” with response option “Yes,” “No,” or “Not sure,” as recommended by the World Health Organization for assessments of vaccine intention.^21^ Participants were not asked directly about receipt of a COVID-19 vaccine as we had anticipated recruitment would finish before vaccine rollout.

Participants were asked “*If I get signs of COVID-19 in the next 4 weeks (cough, sore throat, fever), the following might stop me from getting tested*,” with response options adapted from our previous survey findings,^11,22^ modified to suit the Greater Western Sydney context (e.g., including responses that highlight concerns about visa status). Participants who responded “*I will get tested no matter what*” and listed no barriers were coded as high intention to get tested.

Participants were asked to specify their top three information sources for finding out about COVID-19 in the previous 4 weeks. Risk perception was captured by asking “how serious a problem do you think COVID-19 is currently, in Australia?” with responses ranging from 0 (not serious at all) to 10 (very serious), adapted from our previous COVID-19 surveys.^22^

### Analysis plan

Frequencies were weighted (using poststratification weighting) to reflect each language group's gender and age group distribution (18–29, 30–49, 50–69, and ≥70 years) based on 2016 census data for the Greater Western Sydney population.^19^ All summary statistics presented in the Results section are weighted unless otherwise indicated. A single participant indicated their gender as “other” and was unable to be included in weighted analyses.

Logistic regression models were used to determine factors associated with testing intentions and vaccine willingness, respectively. Supplementary Appendix Table SA7 presents an additional multinomial regression model that examines factors associated with the “not sure” and “no” responses to vaccination compared with “yes” responses; for ease of interpretation, we have combined these into a binary variable (“willing” vs. “not sure/no”) in the main body of the article.

Age group, gender, health literacy, English-language proficiency, years lived in Australia, risk perception, language group, and information sources were included in each model as these have been identified as correlates in relevant research.^7,10^ The regression also controlled for socioeconomic status of area of residence (based on Index of Relative Socioeconomic Advantage and Disadvantage [IRSAD]^23^ deciles by postcode), and whether participants completed the survey before or after June 23rd, when new restrictions were announced for all of Greater Sydney due to a COVID-19 outbreak.^17^

The IRSAD decile was not available for some participants (*n*=5), for example, because they had entered digits that did not correspond to a current or previously valid Australian postcode. IRSAD decile for these participants was replaced with the median IRSAD decile for speakers of the same language in the sample. The Framework for Culturally Competent Health Research^24^ emphasizes the importance of reflecting on whether an analysis may lead to negative and harmful comparison between language groups, potentially reinforcing negative stereotypes or contributing to stigma and discrimination experienced by the community. For this reason, the reference group for language is not identified in regression analyses. Readers can refer to Table 2 to observe descriptive patterns across a language group.

Statistical analysis was conducted using Complex Sample procedures in IBM SPSS Statistics 26. Data are presented as odds ratios (ORs) and adjusted ORs (aORs) with corresponding confidence intervals.

We analyzed a free-text item asking about areas of concern or confusion in the community using content analysis.^25^ Once categories were finalized, O.M. and C.A.B. coded 50 responses, with substantial agreement in coding (Cohen's *κ*=0.95).^26^ Discrepancies were discussed with J.A. before coding the remaining 484 valid responses individually.

## Results

### Sample description

Most participants (88%, *n*=622) were born in a country other than Australia; 31% reported that they did not speak English well or at all (*n*=220); 70% had no tertiary qualifications (*n*=497). Mean respondent age was 45.4 years (95% confidence interval [CI]: 43.9–47.0; range 18–91 years), and 51% were female (*n*=363; Table 1). Inadequate health literacy was identified for 41% of the sample (*n*=290). The most frequently reported information source was official Australian sources or public broadcasters, followed by Australian commercial sources, and social media (Supplementary Appendix Table SA1).

**Table 1.** Descriptive Statistics

Variable	***N***	%
Age group		
18–29	147	20.8
30–49	295	41.7
50–69	193	27.3
>70	72	10.2
Gender^a^		
Male	344	48.7
Female	363	51.3
Language		
Assyrian	133	18.8
Roatian	121	17.1
Arabic	80	11.3
Chinese	76	10.7
Khmer	63	8.9
Dinka	63	8.9
Dari	44	6.2
Spanish^b^	43	6.1
Hindi	42	5.9
Samoan/Tongan	42	5.9
English language proficiency (How well do you speak English?)		
Very well/well	487	68.9
Not well/not at all	220	31.1
Literacy in a language other than English (How well do you read in your main language?)		
Very well/well	589	83.3
Not well/not at all	118	16.7
Adequate health literacy	417	59.0
Highest level of education		
Less than year 12 (less than high school)	115	16.3
Year 12 (high school graduate)	133	18.8
Certificate level I to IV/advanced diploma and diploma level	249	35.2
Bachelor degree level and above	210	29.7
Has a computer with internet access	573	81.0
Has a smartphone	686	97.0
Years living in Australia		
5 Years or less	120	17.0
6–10 years	104	14.7
More than 10 years	398	56.3
Born in Australia	85	12.0
Chronic health conditions^c^		
0	421	59.6
1	154	21.8
2 or more	132	18.6
IRSAD quintile^d^		
1 (Most disadvantaged)	224	31.7
2	131	18.6
3	125	17.7
4	140	19.8
5 (Least disadvantaged)	87	12.3
Total	707	

^a^ One respondent indicated “other/prefer not to say” and is not included in weighted analysis.

^b^ Spanish language group had substantial gaps in recruitment across age groups.

^c^ Chronic health conditions included respiratory disease, asthma, chronic obstructive pulmonary disease, high blood pressure, cancer, heart disease, stroke, diabetes, depression, or anxiety.

^d^ IRSAD presented as quintiles to show overall distribution.

IRSAD, Index of Relative Socioeconomic Advantage and Disadvantage.

### COVID-19 vaccine willingness

Fifty-three percent of participants (*n*=375) said that they would get a COVID-19 vaccine if it was recommended to them, 29.0% (*n*=205) were not sure, and 18.0% (*n*=127) responded that they would not get the vaccine (Table 2). Vaccine willingness (responding “yes”) was 58.4% (*n*=200) for male, and 47.9% (*n*=174) for female participants. This proportion was 56.4% for people with adequate health literacy (*n*=235) compared with 48.0% for people with inadequate health literacy (*n*=139). Across language groups, vaccine acceptance ranged from 29.4% (Samoan/Tongan, *n*=12) to 98.4% (Khmer, *n*=62) (Table 2).

**Table 2.** COVID-19 Testing Intentions and Vaccine Willingness, Descriptive Characteristics

	COVID-19 testing intention		COVID-19 vaccination willingness					
	No barriers		Yes		Not sure		No	
Variable	***n***	%	***n***	%	***n***	%	***n***	%
Age group								
18–29	108	73.8	70	47.7	35	23.7	42	28.7
30–49	236	79.8	169	57.1	83	28.0	44	14.9
50–69	155	80.2	105	54.4	66	34.3	22	11.2
>70	47	65.0	31	42.9	21	29.7	20	27.4
Gender^a^								
Male	262	76.1	200	58.4	90	26.1	53	15.5
Female	284	78.2	174	47.9	115	31.7	74	20.4
Language								
Arabic	65	81.0	52	64.6	8	9.6	21	25.7
Assyrian	106	79.8	54	40.8	27	20.5	51	38.7
Chinese	57	75.5	36	46.8	29	38.7	11	14.5
Croatian	71	58.7	53	43.5	53	43.6	16	12.9
Dari	33	74.4	15	34.4	23	53.1	6	12.5
Dinka	47	74.0	31	48.8	18	28.3	14	22.9
Hindi	42	99.4	34	81.9	8	18.1	0	0.0
Khmer	56	88.9	62	98.4	1	1.6	0	0.0
Spanish^b^	40	93.3	26	59.9	14	32.6	3	7.5
Samoan/Tongan	29	69.0	12	29.4	24	57.6	5	12.9
English language proficiency								
Very well/well	384	78.9	265	54.3	129	26.4	94	19.3
Not well/not at all	161	73.4	110	49.9	77	34.8	34	15.3
Literacy in a language other than English								
Very well/well	452	76.7	305	51.7	189	32.0	96	16.3
Not well/not at all	94	79.5	70	59.3	16	14.0	32	26.8
Health literacy								
Adequate	335	80.3	235	56.4	112	26.8	70	16.8
Inadequate	211	72.7	139	48.0	93	32.2	57	19.8
Years living in Australia								
5 Years or less	91	76.0	75	62.9	24	19.8	21	17.3
6–10 Years	78	75.4	63	61.0	32	30.7	9	8.3
More than 10 years	305	76.7	198	49.8	130	32.7	70	17.5
Born in Australia	71	83.4	37	44.0	19	22.6	28	33.4
Chronic health conditions^c^								
0	338	80.3	239	56.9	78	18.6	103	24.5
1	107	69.3	80	51.5	19	12.4	56	36.0
2 or more	101	76.5	56	42.2	30	22.7	46	35.1
IRSAD (quintile)^d^								
1 (Most disadvantaged)	183	81.6	117	52.1	54	24.1	53	23.8
2	101	77.2	83	63.0	34	25.6	15	11.4
3	78	62.1	57	45.2	43	34.2	26	20.6
4	107	76.2	71	51.1	44	31.2	25	17.7
5 (Least disadvantaged)	77	88.9	47	54.4	31	35.8	9	9.8
Total	546	77.2	375	53.0	205	29.0	127	18.0

^a^ One respondent indicated “other/prefer not to say” and is not included in weighted analysis.

^b^ Spanish language group had substantial gaps in recruitment across age groups.

^c^ Chronic health conditions included respiratory disease, asthma, chronic obstructive pulmonary disease, high blood pressure, cancer, heart disease, stroke, diabetes, depression, or anxiety.

^d^ IRSAD presented as quintiles to show overall distribution.

We observed significant differences in vaccination willingness across language groups (*p*<0.001), controlling for all other covariates (Table 3). Female participants were also more likely to be hesitant about COVID-19 vaccination (aOR=1.63, 95% CI: 1.10–2.43, *p*=0.02), as were participants who did not use Australian commercial information sources as a main way of finding out about COVID-19 (aOR=0.57, 95% CI: 0.37–0.86, *p*=0.01). No other differences were observed across sociodemographic variables.

**Table 3.** Logistic Regression Model Predicting COVID-19 Vaccination Willingness

	Not sure/No (vs. yes willing)			
Predictor	Unadjusted		Adjusted	
	OR (95% CI)	***p***	aOR (95% CI)	***p***
Gender				
Male	Reference		Reference	
Female	1.52 (1.06–2.18)	**0.02**	1.63 (1.10–2.43)	**0.02**
Age group		0.16		0.52
18–29	Reference		Reference	
30–49	0.68 (0.39–1.21)	0.19	0.70 (0.37–1.32)	0.27
50–69	0.76 (0.43–1.36)	0.36	0.58 (0.28–1.19)	0.14
>70	1.21 (0.61–2.39)	0.58	0.62 (0.25–1.55)	0.31
English-language proficiency				
Low	Reference		Reference	
High	0.84 (0.59–1.20)	0.33	0.93 (0.53–1.63)	0.80
Health literacy				
Inadequate	Reference		Reference	
Adequate	0.71 (0.50–1.03)	0.07	0.78 (0.47–1.28)	0.33
Education				
Less than bachelor degree	Reference		Reference	
Bachelor degree or above education	0.89 (0.60–1.32)	0.57	1.09 (0.64–1.86)	0.75
Risk perception	0.95 (0.90–1.01)	0.11	1.00 (0.92–1.08)	0.91
Years living in Australia		0.06		0.18
5 Years or less	Reference		Reference	
6–10 Years	1.08 (0.57–2.06)	0.81	1.43 (0.70–2.95)	0.33
More than 10 years	1.71 (1.03–2.82)	0.04	1.85 (0.97–3.52)	0.06
Born in Australia	2.16 (0.95–4.88)	0.07	2.34 (0.97–5.65)	0.06
Language^a^		**<0.001**		**<0.001**
Information source^b^				
Official Australian source/public broadcaster	0.78 (0.54–1.12)	0.18	1.15 (0.72–1.81)	0.56
Australian commercial source	0.50 (0.34–0.71)	**<0.001**	0.57 (0.37–0.86)	**0.01**
Social media	0.84 (0.59–1.21)	0.35	0.89 (0.59–1.35)	0.59
Friends or family living in Australia	1.43 (0.98–2.10)	0.06	1.15 (0.75–1.76)	0.52
Community	1.10 (0.76–1.59)	0.63	0.95 (0.58–1.55)	0.83
Overseas information source	1.09 (0.76–1.57)	0.63	0.60 (0.34–1.06)	0.08
Chronic health conditions^c^		**0.04**		0.19
0	Reference		Reference	
1	1.24 (0.81–1.89)	0.32	1.33 (0.78–2.29)	0.30
2 or more	1.81 (1.15–2.84)	**0.01**	1.68 (0.95–2.97)	0.08
IRSAD decile^d^	1.01 (0.95–1.08)	0.76	1.02 (0.93–1.11)	0.66

Reference group=“yes” (i.e., willing to get a vaccine); analysis controls for IRSAD and date of survey completion, one respondent indicated “other/prefer not to say” and is not included in weighted analysis.

Bold value indicates *p* values < 0.05.

^a^ Individual comparisons for language group not presented. *p* value represents the main effect of language group.

^b^ Chronic health conditions included respiratory disease, asthma, chronic obstructive pulmonary disease, high blood pressure, cancer, heart disease, stroke, diabetes, depression, or anxiety.

^c^ Information sources entered as separate variables as participants could select more than one.

^d^ *p*-Value for test of model effect includes multinomial regression (both “not sure” and “no” responses to vaccine intention).

Of the 335 participants who were not accepting of the vaccine (no/not sure), almost one third (30.4%, *n*=102) indicated concern about vaccine side effects as their main reason (Supplementary Appendix Tables SA2 and SA3). A further 23.9% (*n*=80) listed safety as a concern, and 11.4% (*n*=38) needed more information before deciding. This ranking was similar across language groups (Supplementary Appendix Table SA2).

Concern about vaccine side effects were also emphasized in free-text responses about aspects of COVID-19 that were concerning or confusing. More than half of these 534 responses voiced concerns about the COVID-19 vaccines (53.0%, *n*=286), with 23.9% (*n*=129) specifically concerned about vaccine safety and side effects (Table 4). Almost one fifth voiced concerns about how COVID-19 information was communicated (17.4%, *n*=94).

**Table 4.** Content Analysis of Areas of Concern or Confusion About COVID-19 in the Community

Area of concern or confusion	Example quote	***N***	%
Information and communication	“Clearer to the point information not an overload; currently there is too much information available that is badly translated and not targeting my community appropriately it is written for highly educated and it is too wordy”	94	17.4
Need for translated information		45	8.4
Need for clear, accurate, and high-quality information		44	8.2
Need for information available in different formats		17	3.2
Vaccines	“Doubts about vaccines. No clarity from government on reactions and side effects. Still unclear about my eligibility for vaccinations. Do we have to take every year like flu injections- no clear information.” “Worried about blood clots post vaccine”	286	53.0
Vaccine safety and side effects		129	23.9
General vaccine questions		86	16.0
Vaccine rollout and practical barriers		58	10.7
Vaccine efficacy		30	5.6
Concerns about AstraZeneca		20	3.8
Other	“The type of Covid-19 and the time it is going to end” “For me, being away from my children is very difficult. I want to know for how long should we remain away from our families”	116	21.4
General covid/pandemic concerns and questions		72	13.4
Need more information about travel bans and border restrictions		30	5.6
Other		15	2.7
No concerns or areas of confusion	“no question as my doctor will inform me on Covid”	95	17.7

One respondent indicated “other/prefer not to say” and is not included in weighted analysis; responses could be coded to more than one area of concern of confusion.

### COVID-19 testing intentions

Three quarters of participants (*n*=546, 77.2%) responded that they would “get tested no matter what” if they developed COVID-19 symptoms in the next 4 weeks (Table 2). This proportion ranged from 58.7% for Croatian speakers (*n*=71), to 99.4% for Hindi speakers (*n*=42). For participants with inadequate health literacy, 72.7% indicated that they would “get tested no matter what” (*n*=211; vs. 80.3% for adequate health literacy; *n*=335).

Intention to get tested for COVID-19 symptoms “no matter what” was significantly associated with age (*p*=0.03), with participants 50–69 years of age less likely to get tested than participants less than 30 years of age (aOR=0.44, 95% CI: 0.20–0.99, *p*=0.048) (Supplementary Table SA4). Intention to get tested also varied significantly across language groups (*p*<0.001). No information sources were significantly associated with testing intention once adjusted for other covariates.

Of the 161 participants who identified at least 1 barrier to getting tested for COVID-19 in the next 4 weeks, the most common barrier to testing was concern about infection at the testing clinic (26.1%, *n*=42), followed by concerns that testing is painful (25.3%, *n*=41) (Supplementary Appendix Tables SA5 and SA6).

## Discussion

### Key findings

This study provides insight into the perspectives of culturally and linguistically diverse Australians during the start of the COVID-19 vaccine rollout, a period characterized by low levels of COVID-19 community transmission (March 21 to July 9, 2021). Three quarters of participants (77%) reported that they would get tested for COVID-19 if they experienced symptoms, “no matter what,” although this proportion varied significantly across language groups. The main barriers to testing were concerns about getting infected at the testing center and about the test being painful.

Just over half (53%) were willing to get a COVID-19 vaccine if recommended to them, one fifth responded they were unwilling, and the remainder unsure, although again, these proportions varied significantly by language spoken at home. Participants were more likely to say “no” or “not sure” to a COVID-19 vaccine if they were female or did not use Australian commercial information sources as a main way of finding out about COVID-19. Key barriers to vaccination were concerns about side effects and safety of COVID-19 vaccines, and a need for more information before making a decision.

We observed higher testing intentions in this study compared with national Australian estimates during a similar time period (77% vs. 45–51%).^5,6^ This study identified different top barriers to testing as in our previous Australia-wide survey in November 2020; although both observed concerns about the test being painful within the top five.^11^ Conversely, we observed lower COVID-19 vaccine willingness in this study compared with national Australian estimates during similar time periods (53% vs. 68–73%),^5,6^ although this rate was similar to a study of 199 people from NSW, Australia, who spoke a language other than English at home (58%),^27^ and higher than a study of 516 Australian refugee and asylum seekers (28%).^27^

Key barriers to vaccination were similar in this study and our November Australia-wide survey (i.e., concerns about safety and needing more information),^7^ although distrust of the government did not feature highly in the current study; only 3% of participants who reported barriers to vaccination said this was because they did not trust the government. This is consistent with findings from a survey of Australian refugees and asylum seekers, in which trust in authorities was not identified as a key COVID-19 concern.^28^

In this study, the main factor associated with testing and vaccination intentions was the language spoken at home, even when controlling for education, socioeconomic status, and English language proficiency. Given existing inequities for people from culturally and linguistically diverse communities, and the lower vaccination intentions observed in this study, public health should prioritize these groups to support testing and vaccination.^14,15^ This approach must work collaboratively with people in these communities to develop tailored, targeted approaches to COVID-19 vaccine communication and rollout.^4,28–30^

In fact, the Australian federal government itself laid out policy in November 2020 emphasizing the need for translated and simple English communication about the vaccines and their rollout, providing ample opportunity for people in these communities to ask questions, working with community leaders and representatives, and embedding interpreter workforce into clinical services.^31^ At the time of study recruitment, this policy had not been adequately implemented in Australia.

However, in response to the growing COVID-19 outbreak in the region, the NSW government demonstrated clear examples of how this policy can be carried out in practice. This included broadcasting non-English versions of COVID-19 press conferences, providing community members opportunity to ask questions directly to government in language, and working with people from culturally and linguistically diverse communities, both members of the public and health services staff, to deliver messages through the government's media channels. Efforts were also made to set up COVID-19 vaccination hubs at churches, mosques, and community centers.

By mid-September 2021, a much higher proportion of Greater Western Sydney adult residents had received their first vaccine dose (ranging 84% to >95%) compared with elsewhere in NSW (82%).^32^ Other studies have also shown that inclusive approaches support COVID-19 testing behaviors, such as community-based strategies implemented in culturally and linguistically diverse neighborhoods in Seattle in the United States.^33^

### Strengths and limitations

The main strength of this study is that we used inclusive recruitment and data collection methods to increase opportunity for participation. This included providing translated versions of the survey, using interpreters, and multiple recruitment avenues. We also included several variables related to culture and language (e.g., English language proficiency, literacy in own language, and years living in Australia), and focused on 10 specific language groups, in an attempt to provide a more nuanced description of the sample that captures some of the complexity within these communities. Limitations are that this study did not ask participants if they had already received the vaccine. State-wide estimates suggest that fewer than 25 per 100 NSW population had received a single dose of the vaccine at the time recruitment closed.^16^ Lastly, we were unable to incorporate specific items about the AstraZeneca vaccine as recruitment began before the news about its side effects hit mainstream media outlets.

## Conclusions

This study of people from culturally and linguistically diverse communities in Greater Western Sydney shows that community members have genuine concerns about COVID-19 testing and vaccination, particularly with regard to safety and wanting more information. Different language groups have unique and specific needs that must be met through local targeted communication strategies. Public health bodies must work collaboratively with communities to provide tailored approaches that support people in these communities to take up COVID-19 testing and vaccination, or risk exacerbating health inequalities further.

## Supplementary Material

Supplemental data

Supplemental data

## Supplementary Material

Supplementary Appendix Table SA1

Supplementary Appendix Table SA2

Supplementary Appendix Table SA3

Supplementary Appendix Table SA4

Supplementary Appendix Table SA5

Supplementary Appendix Table SA6

Supplementary Appendix Table SA7

Supplementary Appendix SA2

## Abbreviations Used

OR: odds ratio
aOR: adjusted OR
CI: confidence interval
IRSAD: Index of Relative Socioeconomic Advantage and Disadvantage
